# Evidence for the Need to Standardize Data Collection on Patient Outcomes Across Adult Day Centers in the United States

**DOI:** 10.1093/geroni/igaa057.2099

**Published:** 2020-12-16

**Authors:** Tina Sadarangani, William Zagorski, Lydia Missaelides

**Affiliations:** 1 New York University, New York, New York, United States; 2 American Senior Care Centers, Nashville, Tennessee, United States; 3 California Association for Adult Day Services, Sacramento, California, United States

## Abstract

Researchers’ ability to measure the impact of adult day centers (ADCs) on participants’ health has been hampered by a lack of large-scale data. We examined categories of data ADCs across the United States are collecting related to patients’ health and health outcomes with the idea of developing a future national cohort of centers. We distributed an electronic survey to ADCs in 50-states on current data collection efforts. Forty states were represented (N=250). Only 32% of ADCs collect patient level data for research and analysis. Vital signs, nutritional risk, falls, and activities of daily living data were most commonly collected. However, validated assessment tools were used in less than 50% of cases. Researchers’ ability to pool data on clinical outcomes among ADC users is limited by lack of data collection and use of uniform outcome measures across ADCs. Standardizing data collection is critical to strengthening ADC programs and demonstrating their effectiveness.

